# Relevance of deep learning to facilitate the diagnosis of HER2 status in breast cancer

**DOI:** 10.1038/srep45938

**Published:** 2017-04-05

**Authors:** Michel E. Vandenberghe, Marietta L. J. Scott, Paul W. Scorer, Magnus Söderberg, Denis Balcerzak, Craig Barker

**Affiliations:** 1 Personalised Healthcare & Biomarkers, IMED Biotech Unit, AstraZeneca, HODGKIN, C/o B310 Cambridge Science Park, Milton Road, Cambridge, CB4 0WG, United Kingdom; 2 Pathology, Drug Safety & Metabolism, IMED Biotech Unit, AstraZeneca, Pepparedsleden 1, 431 50 Mölndal, Sweden

## Abstract

Tissue biomarker scoring by pathologists is central to defining the appropriate therapy for patients with cancer. Yet, inter-pathologist variability in the interpretation of ambiguous cases can affect diagnostic accuracy. Modern artificial intelligence methods such as deep learning have the potential to supplement pathologist expertise to ensure constant diagnostic accuracy. We developed a computational approach based on deep learning that automatically scores HER2, a biomarker that defines patient eligibility for anti-HER2 targeted therapies in breast cancer. In a cohort of 71 breast tumour resection samples, automated scoring showed a concordance of 83% with a pathologist. The twelve discordant cases were then independently reviewed, leading to a modification of diagnosis from initial pathologist assessment for eight cases. Diagnostic discordance was found to be largely caused by perceptual differences in assessing HER2 expression due to high HER2 staining heterogeneity. This study provides evidence that deep learning aided diagnosis can facilitate clinical decision making in breast cancer by identifying cases at high risk of misdiagnosis.

Cancer is an ensemble of diseases with vast molecular diversity between tumours of afflicted patients. In order to maximize the chances of clinical benefit, newly developed cancer treatments are targeted at specific molecular alterations that can be identified in the tumour of each patient prior to treatment initiation^1^. One of the most broadly established approaches to predict targeted treatment efficacy is based on the visual inspection of biomarker expression on tissue sections from a tumour by a pathologist. An example in breast cancer is the semi-quantitative assessment of the expression of the human epidermal growth factor receptor 2 (HER2) as determined by immunohistochemistry (IHC) which defines patient eligibility for anti-HER2 therapies. For patients whose tumour strongly overexpresses HER2, the addition of treatment targeted against HER2 is particularly effective at improving clinical outcome compared to chemotherapy alone^2^. The prevalence of HER2 overexpressing cancers is estimated to lie between 15% and 20%^3^ of the 2.7 million patients diagnosed with breast cancer annually in the world^4^. Accurate assessment of HER2 expression is therefore critical in ensuring patients receive the appropriate therapeutic option. According to the recommendations from the College of American Pathologists and the American Society of Clinical Oncology (CAP/ASCO)^3^, a tumour is determined as HER2 positive if the number of tumour cells displaying strong HER2 overexpression (3+ cells) exceeds 10% of the total tumour population; equivocal if the number of tumour cells displaying moderate HER2 overexpression (2+ cells) exceeds 10% of the total tumour population and negative otherwise (Fig. 1). Patients with positive HER2 status are eligible for targeted therapy, whilst equivocal cases are reflexed to *in situ* hybridization (ISH) testing to determine HER2 status. Negative cases are not considered for anti-HER2 therapy. Significant diagnostic variability has been reported between pathologists^5^,^6^,^7^,^8^,^9^,^10^ and it is inferred that 4% of negative cases and 18% of positive cases are misdiagnosed^7^,^11^. In particular, scoring variability has been shown to be important for cases that show heterogeneous HER2 expression within the tumour cell population^12^,^13^. To ensure diagnostic accuracy, pathologists and oncologists routinely request second opinions. However, second opinions are not always easily accessible and can take several weeks. This situation is likely to become more problematic in the next decade with the increasing number of biomarkers to be evaluated by pathologists for clinical decision making and the shortage of newly trained pathologists^14^.

Computer-aided diagnosis holds great promise to facilitate clinical decision making in personalised oncology. Potential benefits of using computer-aided diagnosis include reduced diagnostic turn-around time and increased biomarker scoring reproducibility. In the last decade, commercial algorithms have been approved by the Food and Drug Administration (FDA) for computer-aided HER2 scoring. Yet, despite evidence that image analysis improves IHC biomarker scoring accuracy and reproducibility in tumours^8^,^10^,^15^, the adoption of computer-aided diagnosis by pathologists has remained limited in practice. This can be explained by limited evidence of added clinical value and by the surplus of time required to predefine tumour regions in the tissue sample^16^. Recently, deep learning techniques have dramatically improved the ability of computers to recognize objects in images^17^ raising the possibility for fully automated computer-aided diagnosis. Among deep learning models, convolutional neural networks (ConvNets) is arguably the most studied and validated approach in a range of image understanding tasks such as human face detection^18^,^19^ and hand-written character recognition^20^. The pathology community is showing increasing interest in deep learning^21^ as demonstrated by studies reporting deep learning based image analysis that can accurately localize cells, classify cells into different cell types^22^,^23^,^24^,^25^ and detect tumour regions within tissues^26^,^27^,^28^,^29^,^30^. Further studies are required to assess the validity and utility of deep learning for clinical decision making.

The objectives of this study were (1) to evaluate the ability of a convolutional neural network (ConvNets) model to automatically recognize cancer cell types compared to classical machine learning techniques, (2) to evaluate the performance of ConvNets to provide accurate HER2 status reviews in clinically realistic conditions and (3) to assess the potential utility of computer-aided diagnosis to facilitate clinical decision making.

## Results

### Deep learning outperforms classical machine learning techniques for cell classification

**Figure 2:**
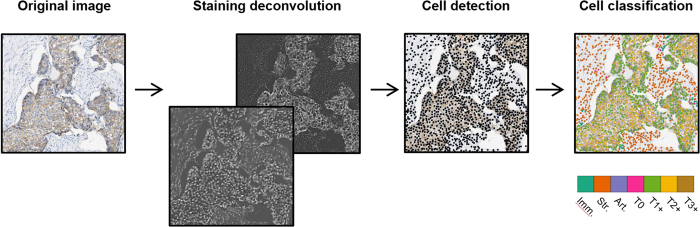
Tumour cells detection and scoring. The original image is deconvolved to generate separate images for haematoxylin staining and HER2 staining; cells are detected using a watershed algorithm; cells are classified using deep learning into either immune cells (Imm.), stroma cells (Str.), artefacts (Art.), tumour 0 cells (T0), tumour 1+ cells (T1+), tumour 2+ cells (T2+) and tumour 3+ cells (T3+).

To automatically score HER2 expression in tumour cells, we propose a simple approach wherein images are first processed to detect cells and machine learning is subsequently used to classify candidate cells into one of the following categories (Fig. 2): stroma cells, immune cells, tumour cells displaying strong HER2 overexpression (3+ cells), tumour cells displaying moderate HER2 overexpression (2+ cells), tumour cells displaying weak HER2 overexpression (1+ cells), tumour cells displaying no HER2 overexpression (0 cells) and artefacts (tissue folds and debris mistakenly detected as cells by image processing). In this section, we describe two classical machine learning approaches as well as the deep learning approach and compare the performance of each approach using 10-fold cross-validation.

A total of 44 image tiles, from a subset of 18 whole-slide images, were included to benchmark the performance of each cell classification algorithm. Image tiles were processed to detect cells using a ruleset implemented in Definiens Developer XD (Definiens AG). A total of 12200 cells were manually annotated (Supplementary Figure 1). For each annotated cell, 18 biologically relevant features were extracted to describe cell morphology, nuclear colour, texture and HER2 membrane staining (Supplementary Table 1). Classical machine learning models were fit using implementations provided in the R programming environment (R Foundation) in order to predict cell type based on cell features. Two models, linear Support Vector Machine (LSVM)^31^ and Random Forests (RF)^32^, were chosen based on their popularity in a number of classification tasks, including cell classification in microscopy images^33^,^34^. In contrast to the two previous models, ConvNets directly learns representations from images, bypassing the need to manually define features. The neural network architecture was designed based on studies by LeCun *et al*.^35^ and Srivastava *et al*.^36^ and is described in more details in the Materials and Methods section. Overall, the model had 466283 parameters that were learned with the backpropagation algorithm in the R programming environment (R Foundation).

Table 1 shows the 10-fold cross-validation performance of the three approaches in terms of F1 scores for each class and overall accuracy. The ConvNets model significantly outperformed LSVM and RF across five of the seven classes and overall (+10% in overall accuracy versus LSVM, two-tailed paired t-test, p = 0.0005;+8% in overall accuracy versus RF, two-tailed paired t-test, p = 0.0001). There was no significant difference in classification performance between ConvNets and either SVM or RF for 2+ cells and 3+ cells.

**Figure 3:**
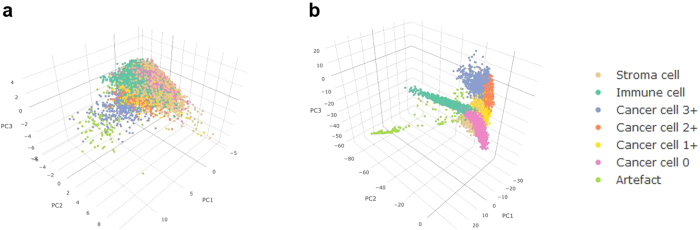
Principal Components Analysis of the hand-crafted and learned features. The scatterplot shows the 3 first principal components values of (**a**) the hand-crafted features and (**b**) the convolutional neural network learned features. Dots correspond to single cells in one cross-validation fold. Colours correspond to manually defined classes.

To better understand the advantage of ConvNets over the classical machine learning models, we investigated the discriminative power of the features utilized in both approaches. Principal Components Analysis was carried-out to project the high-dimensional hand-crafted features and the ConvNets learned features into a more representable 3D space. Figure 3 shows that cells are highly segregated by phenotype in the ConvNets learned features space while cells with different phenotypes are much more overlapping in the hand-crafted features space.

### Deep learning based scoring is substantially concordant with a pathologist scoring

**Figure 4:**
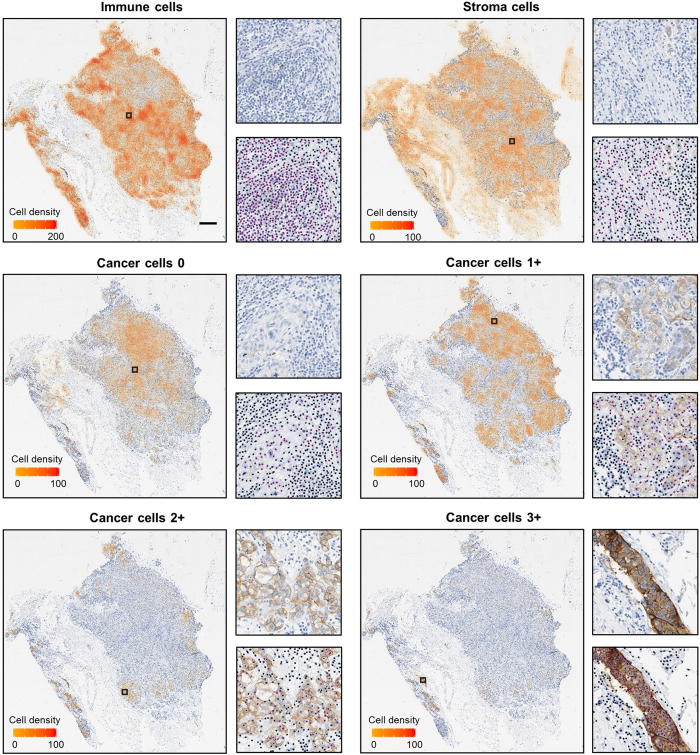
Whole-slide cell classification based on convolutional neural networks. For each phenotype, cell density expressed as the number of cells per 0.015 mm^2^ is shown overlaid with a low resolution view of the original whole-slide image (scale bar: 1 mm). Black squares correspond to areas of high density for each phenotype. A high density area is displayed for each phenotype at full resolution overlaid with the cells classified in that particular phenotype shown as purple dots and the remaining cells shown as black dots.

The HER2 status of 71 confirmed cases of invasive breast carcinoma was determined automatically by deep learning based image analysis and by a pathologist. A pathologist estimated the percentages of 3+, 2+, 1+ and 0 scoring cells within tumour cells. Whole-slide image analysis was performed to detect cells and to classify them using ConvNets (Fig. 4). The percentage of 3+, 2+, 1+ and 0 tumour cells present in the total tumour cell population was calculated for each whole-slide image. Using a standard workstation (16 GB RAM, 6-cores 2 GHz processor), the time required to analyse a typical whole-slide image (27000 × 27000 pixels) was 45 minutes. Finally, a pair of HER2 scores were obtained for each case by applying the CAP/ASCO decision rules^3^ to the tumour cell percentages estimated by the pathologist and obtained via automated image analysis.

The prevalence of HER2 statuses determined by automated image analysis and by the pathologist were, respectively, 62% and 61% for negative cases, 15% and 24% for equivocal cases and 23% and 15% for positive cases. Table 2 reports the agreement between deep learning based scores and the pathologist (the full image analysis outputs and pathologist-based scores for each sample can be found in Supplementary Table 2). Three measures were calculated to evaluate the concordance between automated image analysis and the pathologist: the overall accuracy was equal to 83% (95CI: 0.74–0.92), Cohen’s κ coefficient was equal to 0.69 (95CI: 0.55–0.84) and Kendall’s τ_b_ correlation coefficient was equal to 0.84 (95CI: 0.75–0.93). Removing from analysis the 18 slides that contained some of the 12200 cells used for learning the ConvNet model did not change the level of agreement between automated scoring and the pathologist (overall agreement = 83%, 95CI: 0.73–0.93; Cohen’s κ = 0.69, 95CI: 0.50 – 0.86; Kendall’s τ_b_ = 0.83, 95CI: 0.71–0.95). The concordance between automated image analysis and the pathologist was in the range of previously reported concordance measures between pathologists (Cohen’s κ = 0.56^10^, Cohen’s κ = 0.69^37^ and Kendall’s τ_b_ = 0.69^8^) suggesting that in this cohort, deep learning based scoring provided HER2 scores at least as accurately as a pathologist. Twelve discordant cases were found between the pathologist and automated image analysis. The majority of discordant cases were diagnosed as equivocal by the pathologist but either negative or positive by automated image analysis: 6 cases were scored as equivocal by the pathologist but positive by automated image analysis and 3 cases were scored as equivocal by the pathologist but negative by automated image analysis.

### Deep learning identifies cases at risk of misdiagnosis

**Figure 5:**
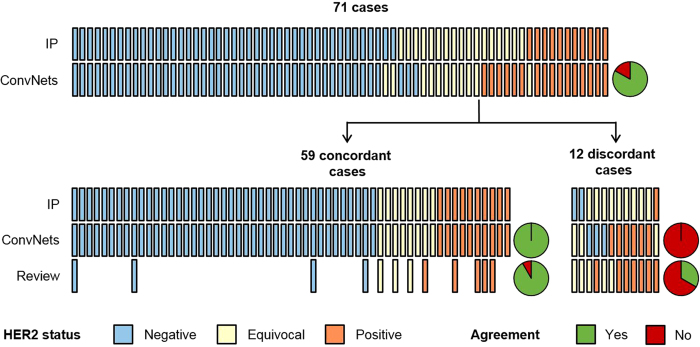
HER2 scoring concordance. Top: comparison between the initial pathologist assessment (IP) and deep learning (ConvNets); bottom-left: comparison between the initially concordant scores and the independent review; bottom-right: comparison between the initially discordant scores and the independent review. Vertical bars represent single cases and pie charts represent overall agreement between either ConvNets or the independent review and the initial pathologist assessment.

Independent scoring of the 12 discordant cases and of 12 selected concordant cases (4 negative cases, 4 equivocal cases and 4 positive cases) was carried out to provide a review of the initial diagnosis for each case (Supplementary Table 3). The review was performed by two experienced HER2 raters blinded to the initial pathologist and deep learning based scores. To prevent intra-rater variability, each case was reviewed a second time after a washout period of several days. Results were averaged across the two sessions and the two reviewers to obtain the review scores. For the cases that were initially concordant between automated image analysis and the pathologist, the review was highly concordant with the initial pathologist assessment (concordance on 11 out of 12 cases; Fig. 5). For the cases that were initially discordant between automated image analysis and the pathologist, the agreement between the initial pathologist assessment and the review was low (concordance on 4 out of 12 cases; Fig. 5). These results indicate that deep learning based review enables to highlight challenging cases which should be carefully assessed to ensure diagnostic accuracy.

**Figure 6:**
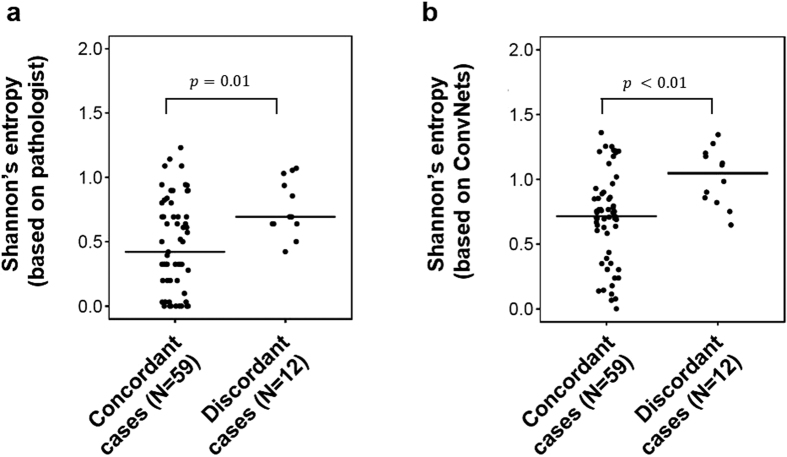
Association of intratumour HER2 heterogeneity with diagnostic ambiguity. Comparison of tumour cells Shannon’s entropy between cases that were consistently scored by the pathologist and deep learning (N = 59) and cases were inconsistently scored by the pathologist and deep learning (N = 12). Shannon’s entropy was calculated either using pathologist defined tumour cell percentages (left) or using deep learning defined tumour cell percentages (right).

After unblinding the scores of each rater, the reviewers investigated each case to identify causes of HER2 assessment variability. Perceptual differences in tumour cell percentage estimation due to widespread HER2 staining heterogeneity was identified as the main driver of disagreement between raters. In most of the ambiguous cases, HER2 staining heterogeneity was caused by commonly occurring technical artefacts and poor tissue quality (Supplementary Figure 2). The effect of HER2 staining heterogeneity on diagnostic ambiguity was confirmed quantitatively. Based on the percentages of 3+, 2+, 1+ and 0 cells defined either by initial pathologist assessment or by automated image analysis, HER2 staining heterogeneity was quantified using Shannon’s entropy, a widely used metric to measure heterogeneity^12^,^38^. HER2 staining heterogeneity was significantly higher for discordant cases compared to concordant cases based on pathologist defined tumour cell percentages (concordant cases: median = 0.42, N = 59; discordant cases: median = 0.69, N = 12; W = 186, p = 0.01, Wilcoxon rank sum test; Fig. 6a) and based on tumour cell percentages defined by automated image analysis (concordant cases: median = 0.71, N = 59; discordant cases: median = 1.04, N = 12; W = 162, p = 0.003, Wilcoxon rank sum test; Fig. 6b).

## Discussion and Conclusion

Historically, the benefit of using computer-aided diagnosis for tissue biomarker was weakened by the shortcomings of image analysis to automatically recognize clinically relevant areas. A growing number of methodological studies are suggesting that the ability of deep learning to achieve complex pattern recognition tasks could lead to a new generation of computer-aided diagnosis tools. However, there is no evidence that deep learning algorithms can assist clinical decision making in oncology. This study constitutes a proof-of-concept that deep learning-based analysis of breast tissue samples enables automated and accurate scoring of a tissue biomarker. It further suggests that computer-aided diagnosis can be instrumental in segregating diagnostically unambiguous from challenging cases that are at risk of misdiagnosis.

Automated tumour biomarker scoring was first assessed by evaluating ConvNets ability to recognize the various cell phenotypes usually present in breast tumour samples. ConvNets achieved significantly higher accuracy compared to SVM and RF, two state-of-the-art classical machine learning algorithms. The accuracy of ConvNets for pattern recognition is generally explained by their capacity to learn abstract representations from training data. Due to the pleomorphy of breast cancer cells^39^, ConvNets are particularly useful to infer discriminatory features that are robust to the diversity of cells within each particular phenotype. A notable exception, however, occurs with 2+ tumour cells and 3+ tumour cells that are distinguishable solely based on membrane staining intensity^3^. ConvNets did not outperform classical machine learning approaches for these two phenotypes, arguably because classical machine learning benefits from the explicit use of membrane staining intensity as a feature. Thus, one approach to reinforce ConvNets performance for HER2 scoring could be to explicitly introduce membrane staining intensity in addition to the trainable features during ConvNet model fitting. An extension of this work would be to evaluate the impact of the training set on ConvNets performance. In particular, the effect of the training set size and the effect of the depth of experience of the operator annotating the training set on ConvNets performance have not been evaluated here and could provide valuable information to optimize the accuracy of cell classification.

The level of agreement between automated scoring and the initial pathologist scoring reported here suggests that once training is performed, the proposed approach can generate valid HER2 scores in a fully automated fashion. Notably, this study did not explore generalizability to samples emanating from various laboratories. Although IHC procedures are becoming increasingly standardised due to the use of automated staining systems, variation in staining intensity can arise between laboratories due to pre-analytical factors (tissue collection and processing). Additionally, differences between slide scanner vendors and slide scanner calibration can affect image intensity levels. Thus, it remains to be determined if and under which conditions a ConvNets model trained in one laboratory can be applied to score slide images generated from different laboratories or if training and validation should be performed separately in each laboratory.

As HER2 heterogeneity has been reported to be a source of inter-pathologist variability^12^,^40^, computer-aided diagnosis could be particularly useful to bring objective and accurate biomarker quantification for these difficult cases. Here, we identified that the discordant cases between automated scoring and the pathologist were significantly associated with HER2 staining heterogeneity. This confirms the confounding role of staining heterogeneity on diagnosis. Among the twelve discordant cases, the independent review established eight cases for which diagnosis differed from the initial pathologist assessment. These results demonstrate that, in this dataset, disagreement between automated scoring and pathologist scoring was mainly due to the presence of ambiguous cases. They further suggests that disagreement between computer-based scores and initial pathologist assessment can be used as an indicator to trigger requests for second opinions. It should be noted that, in this study, case reviews were all performed on digital images whereas the initial diagnosis was performed using the glass-slides with conventional microscopy. Although, studies have reported overall good agreement between diagnoses performed with digital and conventional microscopy^41^,^42^,^43^, the effect of the imaging modality on HER2 status diagnosis was not tested here and could potentially contribute to the observed variability between the initial diagnosis and the review.

In radiology, where computer-aided diagnosis is more widely adopted than in pathology, the usefulness of a computer-aided diagnosis system has been shown to depend not only on the reliability of the information it provides but also on its ability to easily integrate the existing diagnostic workflow^44^. Here, the ability of deep learning to automatically provide biomarker scoring is an important step towards usability of computer-aided diagnosis. In addition, while deep learning is generally associated with prohibitive computing time without the use of a high-performance computing facility, the implementation of the current approach was designed to meet time and computing resources in a typical pathology laboratory. Thus, a choice was made to use time-efficient image processing techniques for cell detection and ConvNets for cell classification. Additionally, the ConvNets model architecture was designed to accommodate realistic memory and computing processor unit specifications. As a result, a regular desktop computer can handle approximately 30 whole-slide images per day enabling the integration of automated HER2 scoring as part of a diagnostic workflow in a high volume pathology laboratory^7^.

Owing to an increasing number of biomarkers that are being quantitatively evaluated to determine patient treatment, cancer diagnosis is becoming more complex and more demanding for pathologists. Thus, increasingly computer-aided diagnosis of tissue biomarkers could become a crucial aspect of ensuring that patients are prescribed the appropriate therapeutic option. The capacity for adoption of automated scoring in the routine clinical workflow is also reinforced by the increasing use of whole-slide imaging scanners in pathology departments. This study specifically focused on the HER2 biomarker yet, it opens the door to the application of deep learning in a larger range of immunohistochemistry biomarkers. In conclusion, this study provides evidence for the accuracy and clinical utility of deep learning-based automated scoring of HER2 in breast cancer.

## Materials and Methods

### Dataset

The dataset consisted of 74 whole-slide images of breast tumour resection samples which either retrieved from the AstraZeneca BioBank or acquired from a commercial provider (Dako Denmark A/S). Slides were obtained by cutting formalin-fixed, paraffin embedded human breast cancer samples into 4 μm-thick sections, stained by IHC for HER2 demonstration (monoclonal Rabbit Anti-Human HER2 antibody, Dako Denmark A/S) and counterstained with haematoxylin using a Dako Autostainer Link48 (Dako Denmark A/S). Slides were digitized with an Aperio ScanScope whole-slide imaging microscope (Aperio, Leica Biosystems Imaging, Inc.) at a resolution of 0.49 μm/pixel. The slides were reviewed to confirm the presence of invasive carcinoma and a total of 71 invasive carcinoma cases were selected for the study.

### Cell detection

Whole-slide images were processed in Definiens Developer XD (Definiens AG) with a custom ruleset. In the first step, tissue was segmented from the background with an automatic threshold operation. Tiles of 2000 × 2000 pixels containing tissue were then extracted from each slide and further processed in parallel. For each tile, colour deconvolution was performed as per Van Der Laak *et al*.^45^ to extract specific channels for the brown HER2 staining and the blue haematoxylin staining from the original colour image. HER2 staining and haematoxylin staining channels were linearly combined into a single image so that pixels belonging to nuclei had negative values and pixels belonging to positive HER2 membrane staining had positive values. The tissue was then segmented into cells using the watershed algorithm^46^.

### Cell classification

A learning set was constructed in order to train machine learning models to classify detected cells into one of the following categories: stroma cells, immune cells, 0 tumour cells, 1+ tumour cells, 2+ tumour cells, 3+ tumour cells or artefacts. First, a set of 44 image tiles were extracted from 18 whole-slide images selected at random from the dataset. Manual annotations were performed within each tile to define regions that contained a unique class of cells. Regions were carefully delineated to contain cells that were as representative as possible of the entire cell type population and to ensure that each cell class was approximately equally represented in the learning set (on average 1600 cells per class).

The first cell classification approach consisted of a classical machine learning workflow: a set of biologically relevant features were extracted for each cell in the learning set and a model was inferred in order to predict the cell’s phenotype given its feature values. In order to extract features, nuclei were first segmented within each cell with an automatic adaptive threshold operation in the haematoxylin channel and positive membrane was detected at the cell borders with a threshold in the HER2 IHC channel. For every detected cell, a total of 18 features, describing nuclear colour, nuclear texture, nuclear morphology, HER2 membrane staining intensity and proportion of positive HER2 membrane staining were extracted (Supplementary Table 1). Feature extraction was performed using Definiens Developer XD (Definiens AG). Two machine learning models were chosen to predict cell type: Support Vector Machines (SVM) and Random Forest (RF)^32^. Both models were fitted using the 18 features extracted from cells detected within the manually annotated regions of the learning set. Each model’s performance was assessed using 10-fold cross-validation. Hyper-parameters for SVM (regularization parameter) and RF (number of features considered per split) were tuned via internal 3-fold cross validation (Supplementary Figure 3). For RF, the number of trees was kept constant at 500 and trees were allowed to grow until each leaf contained only a single class of cells. SVM and RF models were fit with the implementations provided in the *caret* package in R (R Foundation).

The second approach consisted of a convolutional neural network (ConvNets). Compared with other neural networks architectures, ConvNets takes advantage of the spatial structure of images to share weights across units, which therefore limits the number of parameters to be learned and improves rotation, translation and scale invariance. An image patch around each detected cell was extracted and directly used as input to the ConvNets model. A fixed patch-size of 44 × 44 pixels was selected to ensure that even large cells were entirely captured. Given, the image patch, the class of the cell was inferred by passing it through a set of 3 convolution layers followed by fully connected layers. Each convolution layer consisted of 25 learnable 3 × 3 pixels filters. Similarly to Srivastava *et al*.^36^, each convolution was followed with a max-pooling operation and a ReLU activation. The sequence of convolutional layers was followed by a fully connected layer with 500 units and an output fully connected layer with one unit per class. Class probabilities were obtained by applying the softmax function to the output of the last fully-connected layer. A connection dropout probability of 0.5 was added prior to the fully connected layers to prevent from overfitting^36^. Learning was performed by minimizing the training error calculated as the log loss between the inferred class probabilities and ground-truth labels:


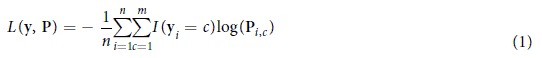


where 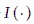 is the indicator function, **y**is the vector of ground-truth classes for cells indexed by *i* = 1…*n* and **P** is a matrix of probabilities such that **P**_*i*_,_*c*_ is the probability of cell *i* belonging to class *c*. The back-propagation algorithm was run over 70 training epochs to minimize the training error. Model performance was compared with RF and SVM using 10-fold cross-validation. The ConvNets model was fit and evaluated within the R programming environment (R Foundation).

### HER2 scoring

The HER2 status for each of the 71 breast carcinoma cases was first determined by a pathologist and by the automated scoring approach based on the CAP/ASCO recommendations^3^. A case was determined as HER2 positive if the number of 3+ tumour cells exceeded 10% of the total tumour population; equivocal if the number of 2+ tumour cells exceeded 10% of the total tumour population and HER2 negative otherwise.

The discordant cases between automated scoring and initial pathologist assessment were reviewed as well as a subset of concordant cases. The concordant cases were selected uniformly across the range of possible HER2 scores (4 negative cases, 4 equivocal cases and 4 positive cases). The reviews were performed by two experienced HER2 raters over two scoring sessions separated by several days for each rater. The reviewers were blinded to the initial diagnosis and the scores from the image analysis. The review score was obtained for each case by averaging the percentages of 3+ cells, 2+ cells and 1+ cells over all the four review sessions and applying the ASCO/CAP rules to the averaged results.

### Statistical analysis

All statistical analyses were performed in the R programming environment (R Foundation). Machine learning model performance was assessed with the overall accuracy as well as F1 scores for each class. The performance of Random Forest, SVM and ConvNets models was evaluated with the overall agreement:


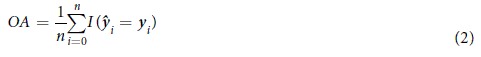


Where **y** is the vector of ground-truth class for each cell and 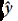 is the class inferred by machine learning. In addition, for each class *c*, the F1 score was calculated as:


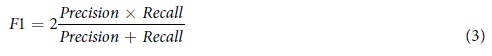


with 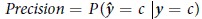 and 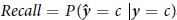.

Cross-validation was performed by creating 10 subsets from 44 image tiles representing 18 cases. Each subset contained either 4 or 5 tiles which were used as the test set uniquely for that iteration and the remaining tiles were used for training. The scores were calculated for each cross-validation iteration and scores were averaged across the iterations. This cross-validation design ensured that cells belonging to the same tile were not used for both learning and testing which could bias performance metrics upward (Supplementary Figure 3). Metrics were compared between ConvNets and either SVM or RF using a two-tailed paired t-test. P-values were not adjusted for multiple comparisons.

The overall agreement, Cohen’s κ coefficient and Kendall’s τ_b_ correlation coefficient were calculated to assess the concordance between automated scoring and the pathologist scores. The overall agreement was calculated as in Eq. 2 but replacing cell classes by HER2 scores. Cohen’s κ is considered to be a more robust estimator as it adjusts the overall agreement with random agreement^47^. Furthermore, it has been previously employed to measure inter-pathologist agreement for HER2 scoring. Kendall’s τ coefficient measures the correlation between ordered categorical variables^48^ and has also been used in inter-pathologist concordance studies. Confidence intervals for each concordance measure were obtained using bootstrap resampling. For each concordance measure, 1000 bootstrap samples were drawn from the original sample with replacement, the concordance was calculated for each bootstrap sample and the 95% confidence intervals of the normal fit to the resampling concordance measure distribution were reported. Similarly to Potts *et al*.^12^, HER2 expression heterogeneity was quantified for each slide with the Shannon entropy^38^ of the distribution of 3+, 2+, 1+ and 0 tumour cells. Shannon’s entropy was compared between concordant and discordant cases with a Wilcoxon rank sum test.

### Code availability

The source code will be made available upon request to the corresponding author.

## Additional Information

**How to cite this article:** Vandenberghe, M. E. *et al*. Relevance of deep learning to facilitate the diagnosis of HER2 status in breast cancer. *Sci. Rep.* **7**, 45938; doi: 10.1038/srep45938 (2017).

**Publisher's note:** Springer Nature remains neutral with regard to jurisdictional claims in published maps and institutional affiliations.

## Supplementary Material

Supplementary Information

## Figures and Tables

**Figure 1:**
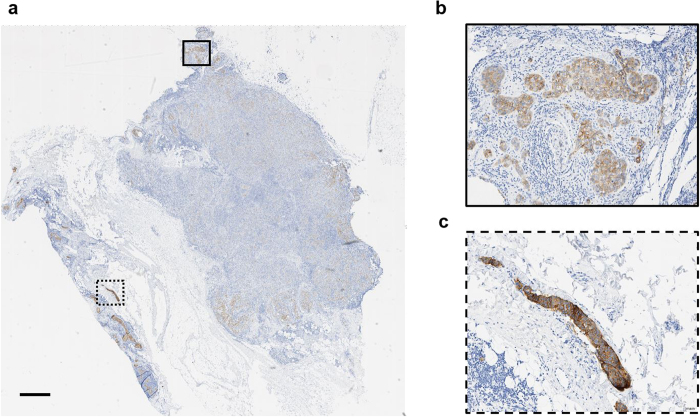
Breast carcinoma HER-2 immunohistochemistry (IHC). (**a**) Low-resolution view of a breast carcinoma tissue section stained by HER-2 IHC (brown) and haematoxylin (blue). The overall HER-2 status for this case has been determined as equivocal by a pathologist and it displays important HER2 staining heterogeneity. Solid line and dotted line rectangles corresponds to areas shown in (**b**) and (**c**), respectively. Scale bar: 1 mm. (**b**) Clusters of tumour cells surrounded by immune infiltration and stroma. The majority of cancer cells display a moderate (2+) HER-2 expression. (**c**) Clusters of tumour cells with strongly positive HER-2 expression (3+) surrounded by stroma.

**Table 1:** Cross-validation (CV) classification performance for each method.

Method	F1 score							Overall Accuracy
	Art.	Str.	Imm.	T0	T1+	T2+	T3+	
Hand-crafted features + LSVM	0.58* ± 0.25	0.64** ± 0.16	0.64** ± 0.23	0.59** ± 0.19	0.66** ± 0.24	0.60 ± 0.23	0.82 ± 0.15	0.68** ± 0.07
Hand-crafted features + RF	0.60* ± 0.32	0.71** ± 0.11	0.66** ± 0.24	0.62** ± 0.18	0.66** ± 0.26	0.58 ± 0.22	0.82 ± 0.16	0.70** ± 0.05
ConvNets	0.72 ± 0.21	0.81 ± 0.15	0.84 ± 0.15	0.74 ± 0.23	0.80 ± 0.11	0.58 ± 0.28	0.78 ± 0.19	0.78 ± 0.07

Abbreviations: Art. (Artefacts), Str. (Stroma cells), Imm. (Immune cells), T0 (0 tumour cells), T1 + (1 + tumour cells), T2 + (2 + tumour cells), T3 + (3 + tumour cells). Two-tailed paired t-test, p-values: * p < 0.05, ** p < 0.01 between ConvNets and either LSVM or RF.

**Table 2:** Confusion matrix between pathologist-based scoring and automated scoring for the seventy-one cases in the cohort.

		Pathologist-based scores		
		Negative	Equivocal	Positive
Deep learning based scores	Negative	41	3	0
	Equivocal	2	8	1
	Positive	0	6	10
	**Overall agreement = 0.83 (95CI: 0.74–0.92)**			
	**Cohen’s κ = 0.69 (95CI: 0.55–0.84)**			
	**Kendall’s τ = 0.84 (95CI: 0.75–0.93)**			

Agreement measures are shown along with their 95% bootstrap confidence intervals.
