# Supplementary material for: Relevance of deep learning to facilitate the diagnosis of HER2 status in breast cancer
Source: Sci Rep. 2017 Apr 5;7:45938. doi: 10.1038/srep45938 (PMC5380996; doi:10.1038/srep45938)
Supplement: Supplementary Information [file srep45938-s1.pdf]

Supplementary information

Relevance of deep learning to facilitate diagnosis of HER2 status in breast cancer

Michel E. Vandenberghe, Marietta L.J. Scott, Paul W. Scorer, Magnus Söderberg, Denis Balcerzak, Craig Barker

CONTENTS

Supplementary Figure 1 2
Supplementary Figure 2 3
Supplementary Figure 3 4
Supplementary Table 1 5
Supplementary Table 2 6
Supplementary Table 3 8

SUPPLEMENTARY FIGURE 1

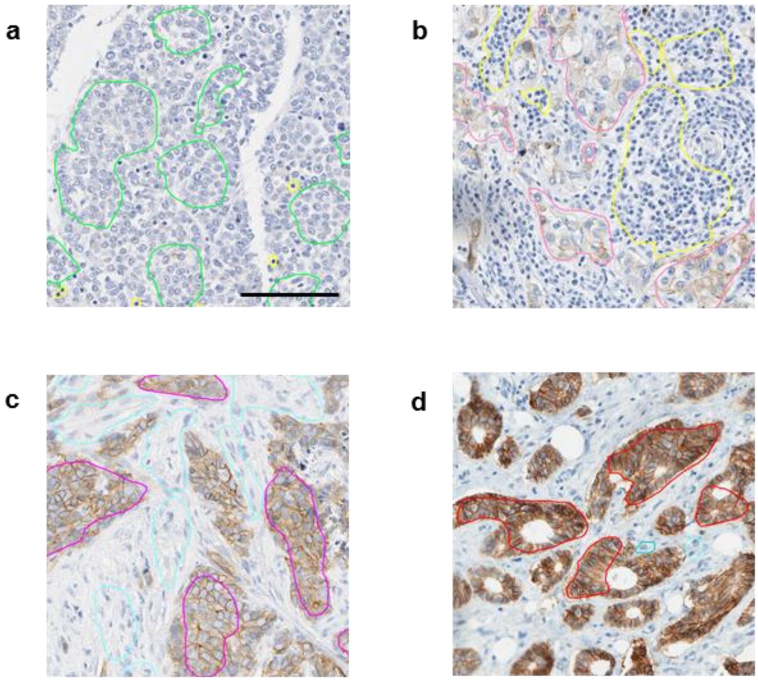

Supplementary Figure 1 – Examples of learning set annotations. 0 tumour cells annotations appear in green (a), 1+ tumour cells annotations appear in pink (b), 2+ tumour cells annotations appear in purple (c), 3+ tumour cells annotations appear in red (d), immune cells annotations appear in yellow (a and b) and stroma cells annotations appear in blue (c and d) (scale bar: 125 µm).

SUPPLEMENTARY FIGURE 2

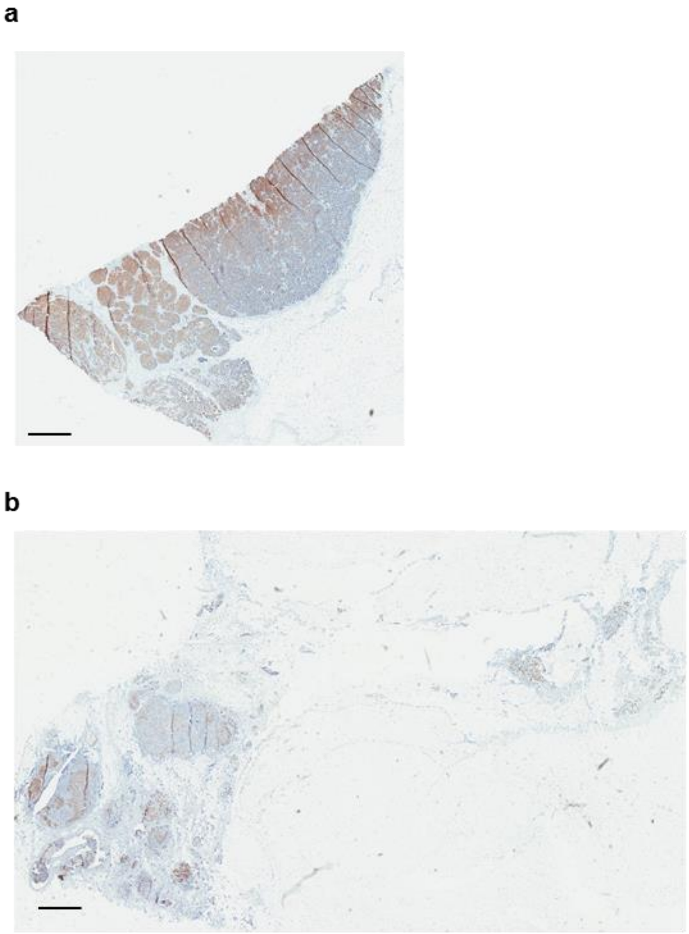

Supplementary Figure 2 – Common patterns of artefactual HER2 staining intensity in breast cancer resection samples. (a) HER2 staining gradient from the upper left border towards the inside of the sample due to incomplete formalin tissue fixation. (b) Poor overall tissue quality.

SUPPLEMENTARY FIGURE 3

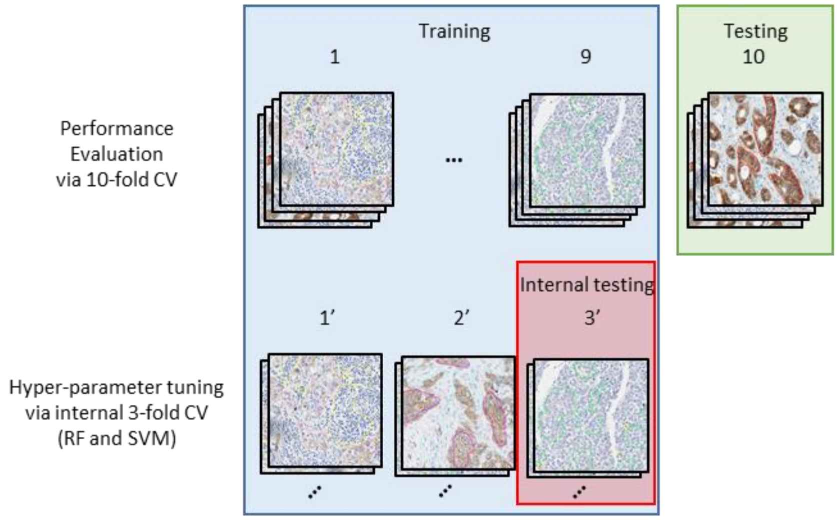

Supplementary Figure 3 – Cross-validation design for model comparison. The top row depicts one iteration of the 10-fold cross-validation employed to compare RF, SVM and ConvNets performance. Each fold contains cells data from 4 or 5 manually annotated image tiles. In each iteration, cells from 9 folds are used for training (blue rectangle) and cells from the remaining fold are used for testing (green rectangle). For each iteration of the 10-fold cross validation, internal 3-fold cross validation was performed to select the best hyper-parameters for SVM (regularisation parameter) and RF (number of features considered at each tree split). The bottom row depicts one iteration of the internal 3-fold cross-validation. Each fold contains one third of the tiles used for training in the external cross-validation iteration, the remaining fold is used to test the performance of the model using a particular hyper-parameter value. The best hyper-parameter values are selected for training the model in the external cross-validation iteration.

SUPPLEMENTARY TABLE 1

List of features for the classical machine learning approaches. Feature description is provided where necessary.

**Feature**	**Description**
Nucleus area (µm)	-
Nucleus circularity	Four times Pi times squared nucleus border length divided by nucleus area.
Nucleus nearest neighbour distance (µm)	Distance from the centre of nucleus to the centre of the nearest nucleus.
Nucleus length/width	Either the ratio of the two eigenvalues of the nucleus pixel coordinates covariance matrix (1) or the ratio of length to width of the nucleus the bounding box (2). The smallest value between (1) and (2) is returned.
Nucleus elliptic fit	Normalized intersection between nucleus and an ellipse with same length and width as the nucleus.
Nucleus shape index	Smoothness of the nucleus border calculate as the border length divided by four-times the square root of its area.
Nucleus mean haematoxylin intensity	-
Nucleus mean red intensity	-
Nucleus mean green intensity	-
Nucleus mean blue intensity	-
Nucleus haematoxylin intensity standard deviation	-
Nucleus red intensity standard deviation	-
Nucleus green intensity standard deviation	-
Nucleus blue intensity standard deviation	-
Number of nuclei fragments	Number of disjoint nucleus objects found within the cell.
N/C ratio	Nucleus area divided by cytoplasm area.
Membrane HER2 intensity	-
Proportion of positive membrane staining	Proportion of the cell membrane which HER2 intensity value is greater than a predefined threshold.

SUPPLEMENTARY TABLE 2

Slide.ID	T0 (%)	T1+ (%)	T2+ (%)	T3+ (%)	Automated scoring	Pathologist scoring
1	0.41	0.53	0.05	0.01	Negative	Equivocal
2	0.12	0.77	0.09	0.02	Negative	Equivocal
3	0.51	0.47	0.01	0	Negative	Negative
4	0.03	0.8	0.16	0.01	Equivocal	Equivocal
5	0.06	0.69	0.2	0.05	Equivocal	Equivocal
6	0.36	0.61	0.03	0	Negative	Negative
7	0.34	0.63	0.03	0	Negative	Negative
8	0.97	0.03	0	0	Negative	Negative
9	0.59	0.4	0.01	0	Negative	Negative
10	0.51	0.48	0	0	Negative	Negative
11	0.99	0.01	0	0	Negative	Negative
12	0.06	0.67	0.25	0.02	Equivocal	Equivocal
13	0.91	0.09	0	0	Negative	Negative
14	0.98	0.02	0	0	Negative	Negative
15	0.12	0.76	0.1	0.01	Equivocal	Equivocal
16	0.3	0.22	0.31	0.18	Positive	Positive
17	0.38	0.21	0.19	0.22	Positive	Equivocal
18	0.29	0.4	0.09	0.21	Positive	Equivocal
19	0.29	0.38	0.04	0.29	Positive	Positive
20	0.06	0.5	0.33	0.11	Positive	Equivocal
21	0.13	0.16	0.15	0.55	Positive	Positive
22	0.06	0.18	0.36	0.4	Positive	Positive
23	0.96	0.04	0	0	Negative	Negative
24	0.31	0.65	0.03	0	Negative	Negative
25	0.64	0.36	0.01	0	Negative	Negative
26	0.48	0.52	0	0	Negative	Negative
27	0.2	0.7	0.09	0.01	Negative	Negative
28	0.48	0.51	0.01	0	Negative	Negative
29	0.1	0.69	0.18	0.03	Equivocal	Equivocal
30	0.15	0.66	0.14	0.05	Equivocal	Negative
31	0.06	0.69	0.17	0.09	Equivocal	Equivocal
32	0.19	0.79	0.02	0	Negative	Negative
33	0.97	0.03	0	0	Negative	Negative
34	0.99	0.01	0	0	Negative	Negative
35	0.34	0.64	0.02	0	Negative	Negative
36	0.62	0.38	0	0	Negative	Negative
37	0.94	0.06	0	0	Negative	Negative
38	0.33	0.61	0.05	0.01	Negative	Negative
39	0.6	0.39	0.01	0	Negative	Negative
40	0.71	0.28	0	0	Negative	Negative
41	0.92	0.07	0.01	0	Negative	Negative

42	0.89	0.11	0	0	Negative	Negative
43	0.55	0.44	0.01	0	Negative	Negative
44	0.26	0.67	0.04	0.03	Negative	Negative
45	0.35	0.31	0.06	0.29	Positive	Positive
46	0.05	0.44	0.17	0.34	Positive	Equivocal
47	0.63	0.36	0.01	0	Negative	Negative
48	0.91	0.08	0	0	Negative	Negative
49	0.06	0.3	0.3	0.34	Positive	Positive
50	0.03	0.78	0.18	0.01	Equivocal	Equivocal
51	0.24	0.75	0.02	0	Negative	Negative
52	0.16	0.65	0.13	0.06	Equivocal	Equivocal
53	0.04	0.36	0.36	0.23	Positive	Positive
54	0.24	0.69	0.05	0.02	Negative	Equivocal
55	0.01	0.7	0.17	0.12	Positive	Equivocal
56	0.02	0.78	0.18	0.01	Equivocal	Negative
57	0.31	0.68	0.01	0	Negative	Negative
58	1	0	0	0	Negative	Negative
59	0.29	0.69	0.02	0	Negative	Negative
60	0.01	0.24	0.41	0.34	Positive	Positive
61	0.06	0.44	0.33	0.17	Positive	Equivocal
62	0.87	0.13	0	0	Negative	Negative
63	0.28	0.69	0.02	0.01	Negative	Negative
64	0.2	0.54	0.22	0.04	Equivocal	Positive
65	0.04	0.3	0.38	0.29	Positive	Positive
66	0.25	0.63	0.08	0.04	Negative	Negative
67	0.89	0.11	0	0	Negative	Negative
68	0.84	0.15	0	0	Negative	Negative
69	0.07	0.18	0.39	0.36	Positive	Positive
70	0.94	0.06	0	0	Negative	Negative
71	0.45	0.53	0.02	0	Negative	Negative

SUPPLEMENTARY TABLE 3

Slide.ID	Automated scoring	Initial path. T0 (%)	Initial path. T1+ (%)	Initial path. T2+ (%)	Initial path. T3+ (%)	Initial path. scoring	Review T0 (%)	Review T1+ (%)	Review T2+ (%)	Review T3+ (%)	Review scoring
1	Negative	0.4	0.4	0.2	0	Equivocal	0.2	0.6	0.2	0.1	Equivocal
2	Negative	0.1	0.7	0.12	0.08	Equivocal	0.2	0.5	0.2	0.1	Equivocal
6	Negative	0.3	0.6	0.1	0	Negative	0.4	0.6	0.0	0.0	Negative
7	Negative	0.1	0.8	0.1	0	Negative	0.4	0.6	0.1	0.0	Negative
8	Negative	1	0	0	0	Negative	0.9	0.2	0.0	0.0	Negative
12	Equivocal	0.15	0.3	0.5	0.05	Equivocal	0.1	0.5	0.2	0.2	Positive
15	Equivocal	0.1	0.4	0.5	0	Equivocal	0.1	0.5	0.4	0.1	Equivocal
16	Positive	0	0.1	0.6	0.3	Positive	0.0	0.3	0.3	0.4	Positive
17	Positive	0.1	0	0.8	0.1	Equivocal	0.0	0.1	0.5	0.4	Positive
18	Positive	0	0.1	0.8	0.1	Equivocal	0.0	0.0	0.5	0.5	Positive
20	Positive	0	0.1	0.8	0.1	Equivocal	0.0	0.1	0.4	0.5	Positive
21	Positive	0	0	0.2	0.7	Positive	0.0	0.0	0.4	0.6	Positive
29	Equivocal	0.1	0.7	0.2	0	Equivocal	0.1	0.5	0.3	0.1	Equivocal
30	Equivocal	0.5	0.5	0	0	Negative	0.2	0.5	0.3	0.1	Equivocal
46	Positive	0	0.5	0.5	0	Equivocal	0.1	0.2	0.5	0.2	Positive
50	Equivocal	0	0.5	0.5	0	Equivocal	0.1	0.3	0.5	0.1	Equivocal
53	Positive	0	0	0.5	0.5	Positive	0.0	0.2	0.3	0.5	Positive
54	Negative	0.5	0.3	0.2	0	Equivocal	0.1	0.5	0.3	0.2	Positive
55	Positive	0	0.8	0.2	0	Equivocal	0.1	0.6	0.3	0.1	Equivocal
56	Equivocal	0.5	0.45	0.05	0	Negative	0.1	0.6	0.3	0.0	Equivocal
61	Positive	0.1	0.3	0.55	0.05	Equivocal	0.1	0.5	0.2	0.3	Positive
64	Equivocal	0	0	0.85	0.15	Positive	0.0	0.1	0.5	0.4	Positive
65	Positive	0	0	0.5	0.5	Positive	0.0	0.1	0.3	0.6	Positive
70	Negative	1	0	0	0	Negative	0.9	0.1	0.0	0.0	Negative
